# Herbaceous plant communities respond more to seasonal precipitation than cumulative drought in the hot deserts of the United States

**DOI:** 10.1111/plb.70083

**Published:** 2025-08-12

**Authors:** T. Ohlert, M. Patton, A. Hallmark, G. Hamilton, S. L. Collins

**Affiliations:** ^1^ Department of Biology University of New Mexico Albuquerque New Mexico USA; ^2^ Department of Biology Colorado State University Fort Collins Colorado USA; ^3^ The Nature Conservancy Santa Fe New Mexico USA

**Keywords:** Aridland, desert, drought, evenness, plant community, species richness

## Abstract

- The hot deserts of the southwestern United States are experiencing increased frequency, severity, and duration of drought due to anthropogenic climate change. Plant communities in these deserts differ in composition, specifically the abundance of annual and perennial species, which could differentiate responses among these ecosystems to drought. Thus, identifying how these desert plant communities respond to prolonged, severe drought is critical to assess vulnerability to climate change. We measured the response of herbaceous plant communities to 4 years of experimentally imposed severe drought in Chihuahuan, Sonoran, and Mojave Desert sites in the southwestern US.
- We imposed year‐round passive rain exclusion treatments with a 66% reduction in ambient rainfall for 4 years at two sites in each of the three US hot deserts. We measured plant species composition and abundance in treatment and control plots during the peak growing season.
- Vegetative cover increased with seasonal precipitation at all six sites. Species richness and evenness varied in response to drought across all sites over the duration of the experiment. At three of the six sites, species richness increased with seasonal precipitation and at three sites species evenness decreased with seasonal precipitation.
- In general, we found that community structure was linked to seasonal precipitation more so than cumulative drought in these herbaceous communities of southwestern US deserts, and that these desert communities are highly resilient following prolonged, extreme drought.

## Graphical abstract

Using a 4‐year drought experiment in the hot deserts of the US, we found that herbaceous plant communities were more sensitive to seasonal precipitation than to the cumulative effects of drought.

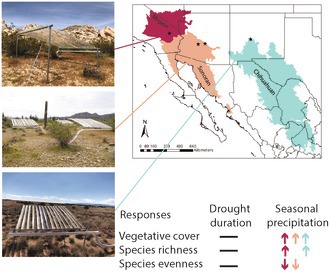

## INTRODUCTION

Climate change forecasts predict increased climate variability and occurrence of climate extremes, including drought (Cook *et al*. 2004, 2018; Dai 2013; IPCC 2014, 2022). Drylands, which cover ~35% of terrestrial ecosystems (Zhou & Yu 2025), are more sensitive to drought and disturbance than mesic ecosystems, making them exceptionally vulnerable to intensifying climatic perturbations caused by anthropogenic climate change (Huxman *et al*. 2004; Burrell *et al*. 2020; Maurer *et al*. 2020). Desert ecosystems, which are limited by water availability (Collins *et al*. 2014), are functionally and structurally different from more temperate ecosystems that experience limitation from light, nutrients, or other resources, more than precipitation (Maestre *et al*. 2016; Hoover *et al*. 2020; Wheeler *et al*. 2021; Berdugo *et al*. 2022). In the hot deserts of the US – Mojave, Sonoran, and Chihuahuan – increased drought frequency and intensity are likely to have long‐term consequences for plant community structure and ecosystem functioning (McAuliffe & Hamerlynck 2010; Gherardi & Sala 2015; Munson *et al*. 2016; Collins *et al*. 2020; Shaw *et al*. 2023). In contrast, deserts of the southwestern US have historically experienced high interannual climate variability, with frequent water limitations, which could result in higher vegetation resilience to drought (Gutzler & Robbins 2011; Maurer *et al*. 2020). Such unique characteristics underscore the need for coordinated cross‐site experiments to better understand how prolonged severe drought will affect the diversity and composition of desert ecosystems in the future.

Although studies on the effects of short‐term drought are somewhat common (e.g., Smith *et al*. 2024), the response of desert plant communities to cumulative, multiyear extreme drought events remains relatively unexamined. While many studies have demonstrated that net primary production (Huxman *et al*. 2004; Shaw *et al*. 2023) and plant species richness (Korell *et al*. 2021; Wheeler *et al*. 2021) are sensitive to changes in precipitation in deserts, prior studies had varied drought length and severity, thus confounding responses across ambient (both dry and wet) conditions. Much less is understood about how desert ecosystems will respond to multiple, consecutive years of severe drought – a climate pattern likely to occur more frequently under climate change. Given that drylands typically experience high interannual precipitation variability and drought frequency, these communities may be highly resilient to prolonged, extreme drought and might return quickly to pre‐drought conditions after the end of a drought period. For example, Wheeler *et al*. (2021) found that Sonoran Desert community structure recovered 1 year after a strong natural drought, indicating that these systems are adapted to periodic, short‐term drought. However, as the frequency of extreme climate events surpasses those in the historic record, desert ecosystems might be particularly vulnerable to multi‐year, extreme drought due to cumulative effects of prolonged water limitation (Cook *et al*. 2018).

The lifespan of species in desert plant communities may affect plant diversity and abundance responses to multi‐year drought. Plant communities in the hot deserts of the US include both long‐lived perennial species and short‐lived ephemerals. Annual plant species, that do not persist in the landscape, are more likely to respond to disturbance (Collins *et al*. 2008; Morris *et al*. 2008; Wilfahrt *et al*. 2021) as their existence is strongly dictated by soil moisture availability (Pickett & Bazzaz 1978; Larson *et al*. 2021). Annuals may employ drought avoidance or bet hedging strategies (Venable 2007; Hallmark *et al*. 2024). Hence, annuals may be highly sensitive to consecutive drought events relative to perennials as they do not maintain above‐ or belowground biomass during droughts. Perennial plants, however, may initially be less sensitive to short‐term drought as they maintain some above‐ and belowground biomass, although their response to drought may increase over multiple years of water stress (Chapin *et al*. 2004; Greaver *et al*. 2012; Slette *et al*. 2022, 2023; Yu *et al*. 2024). These contrasting strategies suggest that the abundance of annuals and perennials within herbaceous communities may determine community responses to prolonged drought. The intershrub zones of the Mojave and Sonoran deserts consist of winter annual species with life spans of just a few months (Brooks 2000). Species of these annual communities can remain dormant in the soil seed bank for many years and only germinate when environmental conditions are favourable (Clauss & Venable 2000; Gremer & Venable 2014). In contrast, Chihuahuan Desert grasslands are dominated by perennial grasses, which make up over 80% of aboveground production (Muldavin *et al*. 2008; Rudgers *et al*. 2018), and therefore productivity will be governed by the sensitivity of perennial grasses to precipitation variability (Báez *et al*. 2013; Munson *et al*. 2013; Collins *et al*. 2020). Thus, we hypothesize that drought should have a more immediate impact on both community structure and ecosystem function of annual communities in intershrub zones in the Mojave and Sonoran Deserts relative to perennial grasslands of the Chihuahuan Desert.

Despite the likely vulnerability of drylands to drought, few studies have experimentally imposed drought in the hottest and driest ecosystems of the southwestern US (Yahdjian *et al*. 2021). We imposed a severe experimental drought for four consecutive years at six sites in these hot deserts (Mojave, Sonoran, and Chihuahuan deserts). We assessed the effects of multiyear drought on vegetation cover, species richness, species evenness, and rank abundance change (Avolio *et al*. 2019). By using identical experimental protocols at each site, we could compare responses of distinct community assemblages, including communities dominated by perennial grasses and those composed of annuals. Our study addressed the following questions. (1) Do impacts of drought on vegetation structure (species abundance, richness, and evenness) increase over consecutive years of drought in desert plant communities? Prior research has shown that richness and abundance of vegetation decreases with drought, and we expected that consecutive years of drought would magnify this response, resulting in fewer species and abundance over the duration of the experiment. (2) Do perennial or annual‐dominated communities incur larger proportional losses of vegetation across multiple years of drought? We expected that communities comprised of mostly annual species would respond more negatively to multiple years of drought, as bet‐hedging strategies mean that many species in these communities avoid drought while persisting in seed banks. (3) Which aspects of desert plant community structure are sensitive to precipitation? Based on previous research, we expect that both species richness and total abundance will be strongly related to seasonal precipitation. In particular, we expected sites dominated by annual species to be more sensitive to seasonal precipitation since annual plant emergence is dependent upon prolonged soil moisture availability and seasonal climate.

## MATERIAL AND METHODS

### Site description

**Fig. 1:**
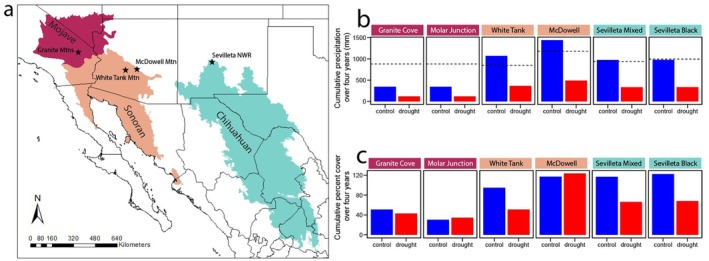
(a) Map of site locations within the hot deserts of the US, and (b) cumulative precipitation (mm) over the 4 years of the experiment in control and drought treatment plots at each site. Dotted line denotes expected cumulative precipitation over 4 years if each year was exactly average (see Table 1 for mean annual precipitation). Due to drought conditions during the experiment, some sites received ambient precipitation well below mean annual precipitation. (c) Cumulative vegetation cover in control and drought treatment plots over the duration of the experiment expressed as the sum of total percentage vegetative cover over 4 years.

We established drought experiments at six sites, two each in Mojave, Sonoran, and Chihuahuan deserts (Fig. 1, Table 1). Although Mojave and Sonoran sites are in desert ecosystems with shrubs and cacti, here we focused on the herbaceous component, which has unique species interactions and phenology. This focus on herbaceous communities in the Mojave and Sonoran deserts also facilitates direct comparisons with the Chihuahuan desert grassland, where woody plants are rare.

**Table 1:** Summary of site information, including biotic and abiotic attributes: mean annual precipitation (MAP), mean annual temperature (MAT), elevation, peak growing season (peak season), total number of unique species in all plots in the pretreatment year (site species), and percentages vegetative cover composed of annuals and perennials in the pretreatment year in the herbaceous communities (% annual, % perennial).

site	desert	MAP (mm)	MAT (°C)	elevation (m a.s.l.)	peak season	site species	% annual	% perennial
Granite cove	Mojave	220	16	1128	Spring	15	100	0
Molar junction	Mojave	220	16	1128	Spring	34	99	1
White tank	Sonoran	212	24	400	Spring	19	100	0
McDowell	Sonoran	295	24	204	Spring	18	100	0
Sevilleta mixed	Chihuahuan	249	14	1669	Fall	20	0	100
Sevilleta black	Chihuahuan	234	14	1669	Fall	9	4	96

Both Mojave sites, Granite Cove and Molar Junction, are in the Granite Mountains Desert Research Center, near Kelso, CA. Granite Cove was established within a *Larrea tridentata* (creosote bush) shrubland, with the herbaceous community dominated by *Schismus barbatus*. Molar Junction was established 0.6 km away in a mixed species shrubland. Abundant species included the annuals *Erodium cicutarium*, *Lotus strigosus*, and *Pectocarya heterocarpa* (Ohlert *et al*. 2021). Mean annual precipitation is 220 mm and mean annual temperature is 16°C. These sites receive most of their precipitation in late winter and early spring, resulting in emergence of the annual community, which typically peaks around early May (Beatley 1969). Both sites are in alluvial fans with sandy, gravel soils.

The Sonoran sites, White Tank and McDowell, are at White Tank Mountain and McDowell Mountain regional parks, respectively, 72 km apart on the west and east edges of the Phoenix, AZ, metropolitan area. These research sites were established as part of the Central Arizona‐Phoenix Long Term Ecological Research project (CAP LTER; Wheeler *et al*. 2021; Shaw *et al*. 2023). Herbaceous communities at both Sonoran sites are dominated by annuals including *Plantago ovata*, *Pectocarya recurvata*, and *Schismus arabicus*. Mean annual precipitation varies from 212 mm at White Tank to 295 mm at McDowell, as the west side of this area receives substantially less precipitation than the east side. Mean annual temperature at both sites is 24°C. White Tank is in a bajada with sandy, aridisol soil, and McDowell is on a mesa, also on an aridisol.

The two Chihuahuan sites, Sevilleta Black and Sevilleta Mixed, are 4.3 km apart in the Sevilleta National Wildlife Refuge, NM, part of the Sevilleta Long Term Ecological Research program (SEV LTER). Both sites are in arid grasslands; the Sevilleta Black site is dominated by the perennial grass *Bouteloua eriopoda* (black grama), and the Sevilleta Mixed site is co‐dominated by perennial grasses *B. eriopoda* and *B. gracilis* (blue grama) with other C4 perennial grasses, including *Pleuraphis jamesii* and *Sporobolus* spp. Mean annual precipitation is 249 mm at Sevilleta Mixed and 234 mm at Sevilleta Black, mean annual temperature is 14°C at both sites. Soils at these sites are Typic Haplargids, with a sandy loam mixture that includes clay and calcium carbonate (Collins *et al*. 2017).

### Experimental design

We established 14 2.5 m × 2.5 m plots at each site, with a permanent 1 m × 1 m vegetation sampling quadrat in the center of each plot. We located plots in inter‐shrub zones, defined as an area not included within the dripline of the shrub canopy. A few plots subsequently included small amounts of woody vegetation and cacti that colonized after the plots were established. Each site included seven unmanipulated control plots and seven plots under rainout shelters. We spatially paired the treatment and control plots in the Chihuahuan sites and randomly assigned them in the Sonoran and Mojave sites. We constructed rainout shelters with frames made of 41.3 mm hollow galvanized steel, ranging from 1 to 1.5 m in height, sloped from south to north to allow water to run down gutters while minimizing shading on plots. We made triangular gutters by bending clear, 11‐cm wide acrylic sheets that allow photosynthetically active radiation (PAR) to pass through (Yahdjian & Sala 2002). We fastened 15 of these to the top of each frame with 5.5 cm between gutters to reduce rainfall by 66% of ambient year‐round. This 66% rainfall reduction was designed to achieve a target 1 in 100‐year annual drought event, given average ambient conditions as in the International Drought Experiment (Lemoine *et al*. 2016; Knapp *et al*. 2017; Smith *et al*. 2024). We erected rainout shelters within a week following pretreatment data collection to ensure consistent year‐round drought treatments (Fig. S1): March 2019 at the Sonoran sites, April 2019 at the Mojave sites, and October 2018 at the Chihuahuan sites.

### Precipitation data

We gathered daily precipitation data for the duration of the experiment using the Multi‐Source Weighted‐Ensemble Precipitation (MSWEP) tool, a global product that reports rainfall extrapolated from satellites and rain gauges (Beck *et al*. 2019). We calculated seasonal precipitation as the amount of precipitation recorded for 120 days prior to the sampling date. This approach accounts for the fact that seasonal precipitation is a better predictor of water conditions in these ecosystems than other time periods of precipitation, such as annual precipitation (Shaw *et al*. 2023).

Over the duration of the 4‐year experiment, ambient seasonal precipitation was occasionally well above normal, e.g., at White Tank and McDowell in the first treatment year where seasonal precipitation was 249 and 332 mm, respectively (Fig. 1b). The duration of the experiment also included an historic natural drought in the region (Williams *et al*. 2022) resulting in some exceptionally dry conditions in control plots. For example, the Mojave sites received just 3 mm precipitation in the preceding 120 days before the fourth year of data collection and just 18 mm in the third year of the experiment. These exceptionally dry ambient conditions resulted in many years in which precipitation in control plots was well below the planned 66% reduction from the long‐term average.

### Data collection

We identified each species rooted within each quadrat and visually estimated abundance as percentage ground cover in the 1 m × 1 m quadrat for each species. Percentage cover used a continuous scale from 0.1% to 100% using only whole numbers for cover >1%. We chose data collection dates to coincide with peak biomass of each ecosystem. As the majority of rainfall is in winter and spring, peak biomass in the Mojave and Sonoran sites occurs in spring, when soils are moist and temperatures are favourable (Wheeler *et al*. 2021; Shaw *et al*. 2023). In the Chihuahuan sites, a heavy monsoon season during July and August results in a peak biomass season in autumn (Muldavin *et al*. 2008; Notaro *et al*. 2010). We therefore collected pretreatment community composition data in March 2019 at the Sonoran sites, April 2019 at the Mojave sites, and October 2018 at the Chihuahuan sites. We sampled vegetation composition annually at peak standing biomass for the next 4 years of the experiment.

### Community structure

We calculated total cover by summing cover of each species within each quadrat. We classified species as either annual or perennial, excluding shrubs and cacti, and calculated cover as the sum of cover from all species classified as annuals or perennials. Species richness was defined as the number of unique species identified in each 1 m × 1 m quadrat. We calculated evenness using the EQ index which accounts for similarity of abundances between species based upon a rank‐abundance curve and is independent of species richness (Smith & Wilson 1996). We used the codyn package in R (v. 2.0.5;Hallett *et al*. 2016; Avolio *et al*. 2019) to quantify species gains as the number of new species in a plot from the previous year divided by the total number of unique species in both the current and previous year. Similarly, we quantified species losses as the number of species present in the previous year but not present during the current year, divided by the number of unique species in both years (Hallett *et al*. 2016; Avolio *et al*. 2019). Rank change is a measure of reordering of abundances among species in a plot. We again used the codyn package in R to calculate rank change as the absolute value of the average change in species ranks between current year and previous year for each replicate, divided by the total number of unique species in both time periods (Hallett *et al*. 2016; Avolio *et al*. 2019).

### Statistical analyses

We measured effect size using the Relative Interaction Intensity index (RII):

$$\frac{t-c}{t+c}$$

where, *t* is the value of a community property in a drought treatment plot, and *c* is the value of that community property averaged across 5 years of control plots (pretreatment year +4 years during treatments; Armas *et al*. 2004). We averaged the abundance of control plots to account for interannual variability in ambient conditions although some sites experienced conditions throughout the study that were drier or wetter than average (Fig. 1b). This method creates a stable reference point that does not change from year to year. RII was thus calculated for each treatment plot in each year for five metrics of community structure: total cover, species richness, species evenness, perennial cover, and annual cover. RII is bounded between −1 and 1 and can incorporate situations in which the community values are 0, unlike other indices, such as log response ratio. The latter property of RII is especially important in low‐productivity desert plant communities which often have no seasonal growth, especially under extreme drought. We used the qt function in the stats package (v. 4.1.2; R Core Team 2021) to generate 95% confidence intervals for RIIs, and we considered responses to treatment as significant when the 95% confidence interval did not overlap 0.

To test whether drought effects on community metrics increased over the duration of the experiment, we created separate models for each site, testing the impact of drought treatment, the impact of experiment year, and the treatment by year interaction for three metrics (vegetative cover, species richness, species evenness) in the form of metric ~ treatment × experiment year. Plot was used as a random effect along with year in an autocorrelation structure to account for repeated measures of the same plots over time. In this model, the treatment by experiment year interaction term is the informative term for determining whether impacts of the drought treatment change over time.

We also tested whether communities changed more in drought or control plots at each site over the 4‐year experiment. We calculated change in richness and evenness as well as rank abundance change from the pretreatment year until year 4 for each plot using the RAC_change function in the codyn package (Avolio *et al*. 2019). We then created a separate model including all sites for each of the three metrics of change in the form of change metric ~ treatment × site. We then used the emmeans function in the emmeans package (v. 1.10; Lenth 2025) with the pairs function in the graphics package (v. 4.1.2; R Core Team 2021) to create contrasts of drought and control treatments for each site.

To test sensitivity of community metrics to precipitation regardless of treatment year, we created independent linear mixed effects models for each site and each of three community metrics (total cover, species richness, species evenness) using plot as a random effect with year in an autocorrelation structure to control for repeated measures over time. Sensitivity to seasonal precipitation was considered significant when the slope of the regression line had a *P*‐value < 0.05. For these analyses, we included both control and treatment plots in the same regression. Due to historically dry ambient conditions in some treatment years, conditions in control plots were sometimes drier than conditions in drought plots of wetter years.

We used R Statistical Software (v. 4.1.2; R Core Team 2021) for all analyses and the tidyverse (v. 1.3.1; Wickham *et al*. 2019) and plyr (v. 1.8.6; Wickham 2011) packages for data manipulation and visualization. The nlme package was used for mixed effects models (v. 3.1‐166; Pinheiro *et al*. 2024). This work was completed as part of the lead author's doctoral dissertation (Ohlert 2022).

## RESULTS

### Ambient and experimentally manipulated precipitation

Ambient precipitation over the duration of the study included many extreme dry years for some sites which resulted in remarkably dry conditions in drought treatment plots. The Mojave sites, Granite Cove and Molar Junction, received just 53, 67, and 93 mm in the second, third, and fourth years of the study compared to a mean annual precipitation of 220 mm (Table 1, Table S1). As these conditions resulted in a combined ambient rainfall <50% of expected over the 4‐year study (Fig. 1b), drought treatment plots received just 118 mm of rainfall cumulatively across all 4 years of the study, and in the driest year received just 8% of mean annual rainfall. Sonoran sites, White Tank and McDowell, had similarly dry conditions in 2021, receiving 203 mm and 186 mm of precipitation, respectively, but wet years in 2020 and 2023 resulted in precipitation received over the 4‐year study above average for these sites. The Sevilleta sites in the Chihuahuan Desert received almost exactly average precipitation over the 4 years of the study (243 mm at Sevilleta Mixed and 244 mm at Sevilleta Black; Fig. 1b), but this was attributable to dry conditions in 2022 balanced by wet conditions in 2020.

### Community responses

**Fig. 2:**
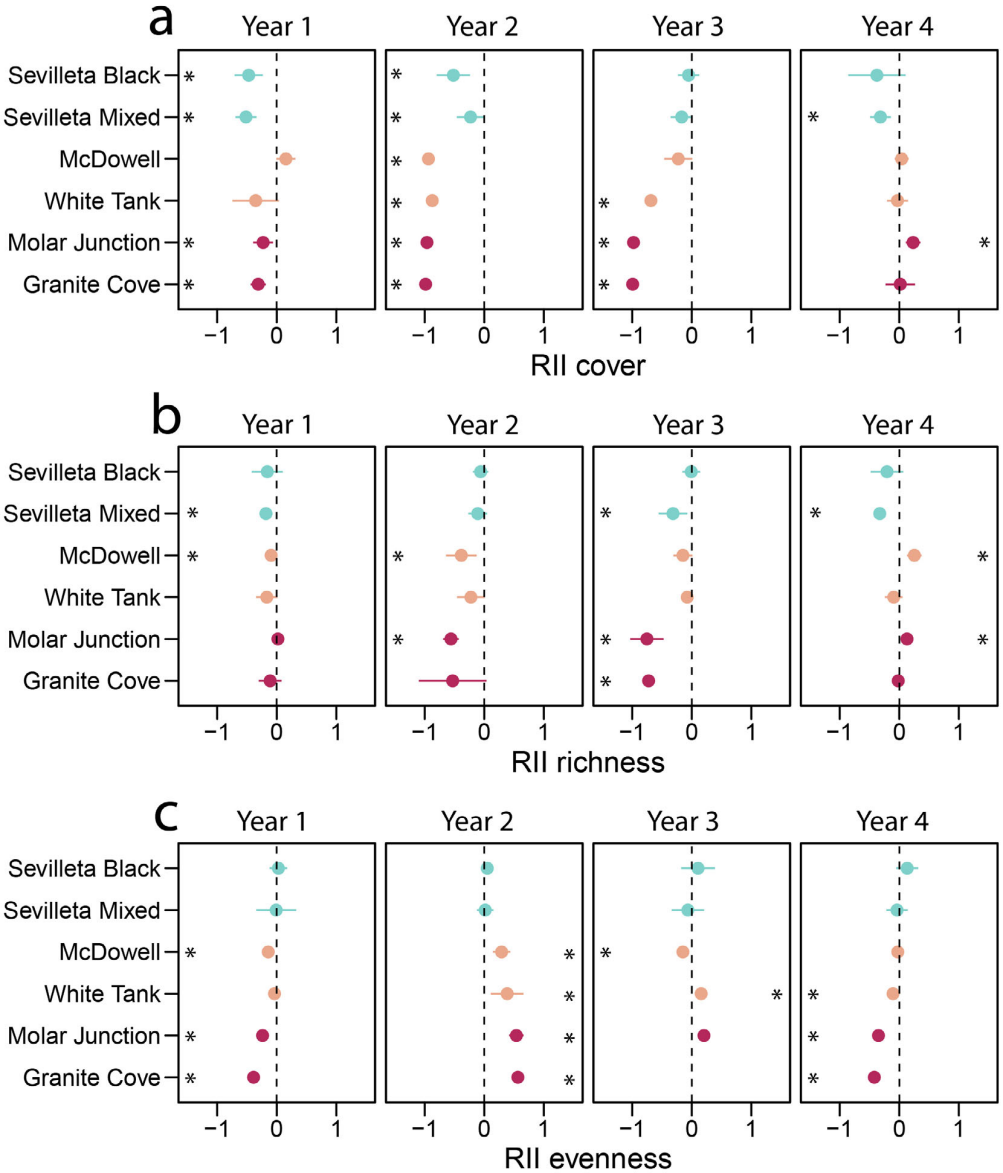
Relative Interaction Intensity index (RII) for: (a) total vegetation cover, (b) species richness, and (c) species evenness in six desert plant communities in the US hot deserts for 4 years of drought treatment. Mean responses are shown with points and error bars are 95% confidence interval. Asterisks denote significant responses in which 95% confidence intervals do not overlap 0. Mean values and confidence intervals are shown in Table S2.

Total vegetative cover decreased in response to drought at four sites in the first year, all six sites in the second, three sites in the third, and one site in the fourth year (Figs. 1c and 2a, Table S2). However, at the Molar Junction site in the Mojave Desert, cover in drought treatment plots was higher than the long‐term average in year 4 when precipitation was ~50% of average (Fig. 2a, Table S2). Species richness decreased at two sites in the first and second years, three sites in the third year, and one site in the fourth year (Fig. 2b, Table S2). Richness slightly increased in the fourth year at both the Molar Junction site in the Mojave Desert and the McDowell site in the Sonoran Desert compared to the long‐term average (Fig. 2b, Table S2). No site showed consistent changes in species richness across years. Evenness decreased at three sites in the first and fourth years, decreased at one site in the third year, and increased at four sites in the second year and one site in the third year (Fig. 2c, Table S2). Note that in the third year, precipitation was so low that the Mojave Desert sites did not have enough replicates to calculate confidence intervals for evenness as most plots contained only one species. No site exhibited consistent changes in species evenness over the entire study period.

**Fig. 3:**
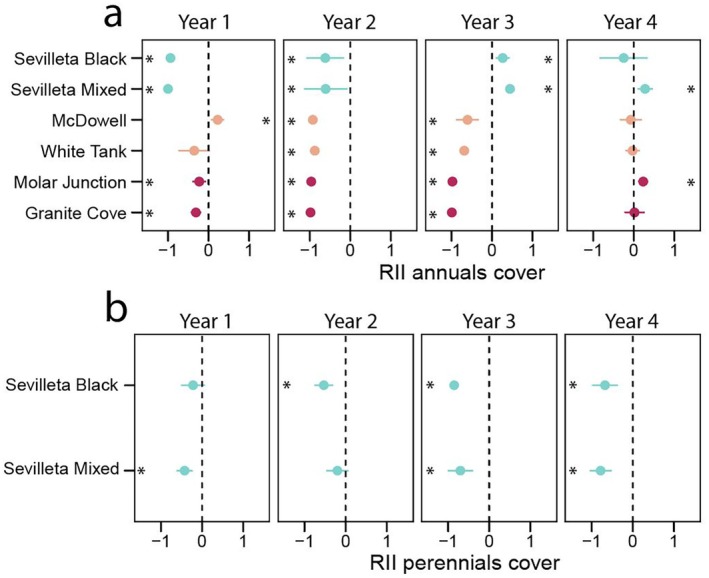
Relative Interaction Intensity index (RII) for the cover of (a) annual species, and (b) perennial species in six desert plant communities in the US hot deserts for 4 years of drought treatment. Mean responses are shown with points and error bars are 95% confidence interval. Asterisks denote significant responses in which 95% confidence intervals do not overlap 0. RII was not calculated for perennial species in the Mojave and Sonoran Deserts in either treatment year due to a lack of data. Mean values and confidence intervals are shown in Table S2.

Cover of annual plants decreased in drought plots at four sites in the first and third years, all six sites in the second year, and none in the fourth year (Fig. 3a, Table S2). Conversely, annual cover increased at one site in the first year and two sites in the third and fourth years (Fig. 3a, Table S2). Perennial plants were present only at the two Chihuahuan Desert sites. Cover of perennial plants decreased in drought treatment plots at Sevilleta Mixed in the first year, at Sevilleta Black in the second year, and at both sites in the third and fourth years (Fig. 3b, Table S2).

### Cumulative effects of 4 years of drought treatment

**Fig. 4:**
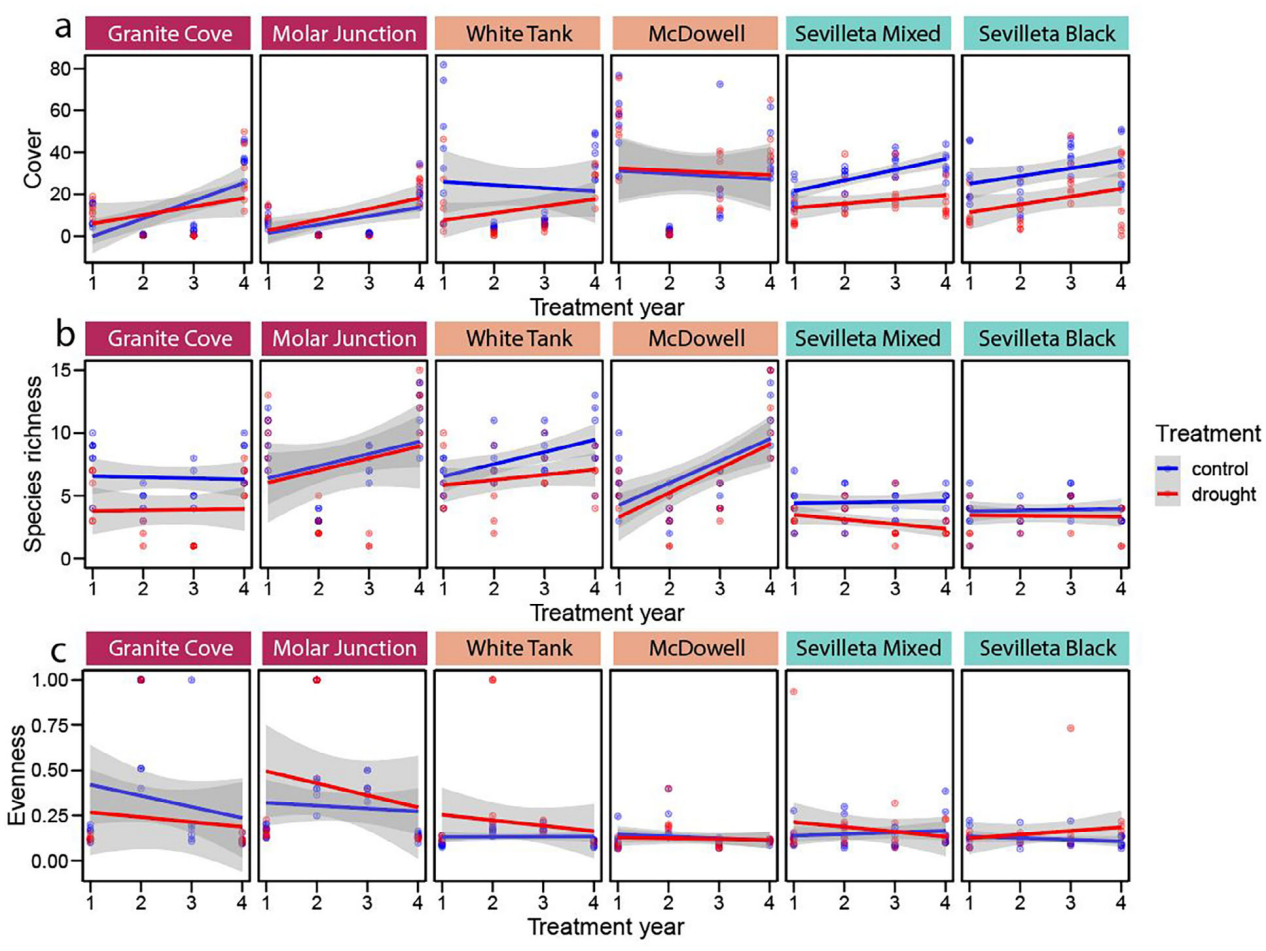
Correlations with treatment year of (a) vegetation cover, (b) species richness, and (c) species evenness for each treatment separated by site. Year × treatment interactions were not significant for any site or metric. Results are presented in Table S3.

The cumulative effects of drought treatment on community metrics over 4 years were evaluated by assessing the interaction between treatment and experiment year. In most cases, the drought treatment did not alter community responses relative to fluctuations seen in control communities experiencing ambient conditions over the study period (i.e., slopes of regressions over time did not differ between treatments). The effect of drought treatment on cover did not change over time at any site, although it decreased marginally at Sevilleta Mixed (*P* = 0.07; Fig. 4a, Table S3) and Granite Cove (*P* = 0.09; Fig. 4a, Table S3). No treatment × experiment year interactions were observed for species richness at any site (Fig. 4b, Table S3), and the interaction effect on evenness was only marginally significant at Molar Junction (*P* = 0.08; Fig. 4c, Table S3). Similarly, community metrics between treatment and control plots changed at some sites from pretreatment to the fourth year of the experiment. Species richness declined more in drought treatment plots in year 4 relative to pretreatment at Sevilleta Mixed (*P* = 0.002; Table S4) and marginally at Granite Cove (*P* = 0.07; Table S4), while changes in evenness did not differ by treatment at any site. Rank changes were larger in drought treatment plots only at Sevilleta Mixed (*P* = 0.04; Table S4).

### Sensitivity to seasonal precipitation

**Fig. 5:**
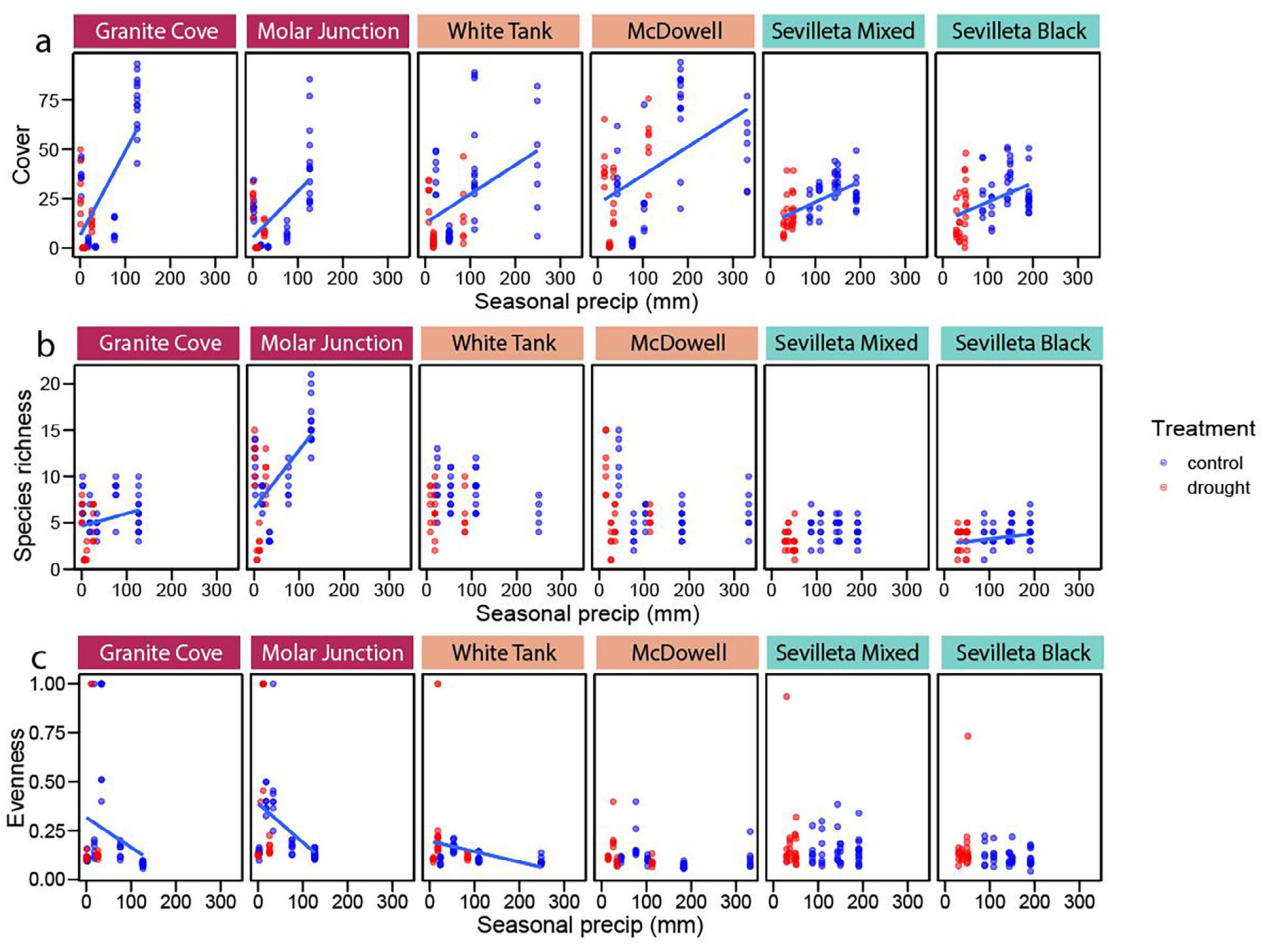
Correlations with seasonal precipitation of (a) vegetation cover, (b) species richness, and (c) species evenness for each treatment separated by site. Regression lines denote instances in which seasonal precipitation was a significant predictor of the metric. Further results are provided in Table S5.

Total cover at all six sites was positively related to seasonal precipitation, with the relationship ranging from 0.4% mm^−1^ at Granite Cove to 0.1% mm^−1^ at Sevilleta Black (Fig. 5a, Table S5). Species richness was correlated with seasonal precipitation at just three sites, and was positively related at Granite Cove, Molar Junction, and Sevilleta Black (Fig. 5b, Table S5). At Granite Cove and Sevilleta Black, species richness increased by ca. 1 species for every additional 100 mm precipitation, while Molar Junction added about 6 species over the same increase in precipitation (Table S5). Evenness declined significantly with precipitation at three sites, Granite Cove (*P* = 0.04), Molar Junction (*P* = 0.002), and White Tank (*P* = 0.04), and marginally at McDowell (*P* = 0.07) and Sevilleta Black (*P* = 0.08; Fig. 5c, Table S5).

## DISCUSSION

We evaluated the effects of a 4‐year extreme drought on the structure of herbaceous plant communities in the hot deserts of the southwestern US. Contrary to our expectations, drought effects on plant communities were rarely cumulative, but were strongly influenced by precipitation in the growing season, irrespective of previous year's rainfall. Strong regional drought during the study period resulted in extremely dry ambient conditions which magnified the drought treatment effects, especially in the Mojave Desert. Overall, vegetative cover, species richness, and species evenness were more strongly related to seasonal precipitation than to duration of the drought treatment. As aridity and instances of drought in the southwestern US. are expected to increase this century due to climate change, herbaceous plant communities of these deserts will likely experience lower vegetation cover and species richness.

### Decline of perennial species in the Chihuahuan Desert

Our results add to a growing body of evidence showing that desert communities dominated by perennial grasses are particularly sensitive to prolonged drought. Perennial cover in the Chihuahuan Desert was consistently negatively impacted by the drought treatment across the duration of the experiment, consistent with previous studies that documented the sensitivity of dominant perennial grasses in this region to prolonged dry conditions (Báez *et al*. 2013; Ladwig *et al*. 2014; Collins *et al*. 2020; Yu *et al*. 2024). The response of annual species in the Chihuahuan Desert shifted from negative to positive over the duration of the treatment, which might suggest that subdominant annual species in these ecosystems are co‐limited by water availability and competition with dominant perennials (Ning *et al*. 2024). This dynamic between dominant perennial grasses and subordinate species has been documented in previous studies in the Chihuahuan Desert (Peters & Yao 2012). While decreases in the abundance of dominant species may promote subordinate species, some evidence suggests that drought can reduce the growth of dominant species without significantly affecting overall plant diversity (Báez *et al*. 2013). However, dominant grasses play a critical role in ecosystem function, and their loss cannot easily be compensated for by subdominant species (Smith & Knapp 2003). As drought frequency increases in the Chihuahuan Desert, the negative impacts on dominant perennial grasses are likely to reduce vegetative cover, increase the size of areas of unvegetated soil, and lead to a reordering of dominance, from perennial grasses to shorter‐lived species.

### Seasonal precipitation altered community structure

Community structure was generally sensitive to seasonal precipitation, especially total cover which was sensitive to precipitation at all six sites, and this consistent sensitivity to water availability is well documented (Huxman *et al*. 2004; Maurer *et al*. 2020). Annual forbs play a novel role in some desert ecosystems through delayed germination while awaiting favourable abiotic conditions, often when seasonal rainfall is above a threshold for germination (Cayan *et al*. 1999; Venable & Pake 1999; Bowers 2005; Venable 2007; Gremer & Venable 2014). Species richness positively correlated with seasonal precipitation at the two Mojave Desert sites and one Chihuahuan Desert site. Although recent research in temperate grasslands of North America shows that drought manipulation might have no effect on species richness (Castillioni *et al*. 2020; Batbaatar *et al*. 2021), our results are similar to those of a recent meta‐analysis, demonstrating that drought effects on species richness are highest in the more arid environments (Korell *et al*. 2021). Loss of aboveground vegetation will likely incur a commensurate decline in richness (Mittelbach *et al*. 2001) and, therefore, much of the declines in species richness observed here may be attributable to loss of total vegetation cover. Similar drought manipulation studies in annual communities of Mediterranean shrublands found that drought did not directly affect species richness (Tielbörger *et al*. 2014), but changed the slope of the relationship between species richness and aboveground net primary production (Alon & Sternberg 2019).

Previous studies on drought impacts on evenness reported either no effects (Batbaatar *et al*. 2021) or positive effects (Alon & Sternberg 2019; Castillioni *et al*. 2020). Dominant species are known to be particularly important drivers of ecosystem function in response to drought (Báez *et al*. 2013; Hoover *et al*. 2014; Knapp *et al*. 2020; Smith *et al*. 2020), therefore, increased evenness and a commensurate decrease in dominance might suggest an overall decline in ecosystem function. Aboveground production in the Chihuahuan Desert is driven by dominant perennial grasses, and the herbaceous communities of the Mojave and Sonoran Deserts are dominated by productive, non‐native annual grasses (Ohlert *et al*. 2021; Wheeler *et al*. 2021). Consequently, this dynamic of drought‐induced increased evenness, and thus decreased dominance, could inhibit the spread of non‐native species in the Mojave and Sonoran Deserts while impairing native dominant species in the Chihuahuan Desert that are critical for ecosystem functioning (Rudgers *et al*. 2018; Collins *et al*. 2020). Thus, increases in evenness could have divergent ecological consequences, impacting ecosystem function by constraining invasive species in some regions, while impairing key native species in others.

### Lack of cumulative effects after passive manipulation

One potential explanation for the lack of cumulative effects observed in our study is that annual communities, such as those of the Mojave and Sonoran Deserts, may be less sensitive to abiotic and biotic factors that typically drive cumulative drought effects. Previous studies suggest that community changes in response to perturbations increase over time (Smith *et al*. 2009; Seabloom *et al*. 2021). As perturbations occur over consecutive years, their impacts on communities often accumulate (Felton *et al*. 2021), and in the context of drought, this accumulation may be driven by abiotic factors, such as soil moisture influenced by precipitation from previous years (Cook *et al*. 2009; Sala *et al*. 2012), and biotic factors, such as litter accumulation (Chen *et al*. 2024) or cumulative physiological stress (Vandegeer *et al*. 2020). In the Sonoran Desert, for example, annual communities show stronger associations with recent precipitation than with precipitation from the previous season, with aboveground net primary production (ANPP) and taxonomic diversity more closely tied to current‐year precipitation rather than lag effects from prior years (Wheeler *et al*. 2021; Shaw *et al*. 2023). Not only are these annual plant communities short lived, but they are also adapted to stochastic resource availability.

The historically dry ambient conditions in the southwestern US during the second and third years of the experiment (mid‐2020 through 2022) likely contributed to the lack of cumulative effects of drought observed in this study. Extreme drought in the middle years, followed by less extreme conditions in the fourth year, may have created modal dynamics in community metrics, making it difficult to capture clear cumulative effects over the course of just 4 years. For instance, at the McDowell site in the Sonoran Desert, the highest species richness was recorded in the first and fourth years for both control and treatment plots, suggesting that treatment effects had little persistent influence on species richness throughout the experiment. This highlights a limitation of the passive manipulation approach used in this experiment and in the broader International Drought Experiment. Passive manipulation is often employed in drought studies due to its cost‐effectiveness, as active control of precipitation requires substantially more infrastructure (Fraser *et al*. 2013; Knapp *et al*. 2017). Although passive manipulation ensures consistent comparisons across sites under near‐average conditions, the inclusion of more sites in cross‐site comparisons increases the likelihood that some sites will experience rainfall far outside the historical mean. Additionally, as the duration of the experiment increases, the likelihood of encountering precipitation conditions well outside of historic average at more sites also rises.

## CONCLUSION

Our cross‐site experimental drought treatments demonstrate that drought impacts on desert plant communities in the southwestern US are influenced more by annual precipitation than by the cumulative effects of prolonged drought. While drought generally reduced plant cover and species richness, the variation in seasonal rainfall played a key role in shaping these responses. In the Chihuahuan Desert, perennial grasses consistently declined during the drought years, while annual species increased in abundance once perennial cover was reduced. This shift suggests that annuals and perennials compete for resources in this system and drought can favour annual species. In the Mojave and Sonoran Deserts, we did not see clear cumulative drought effects, likely because of the irregularity of rainfall patterns during the study. However, cover and richness at several sites rebounded with higher rainfall after several years of drought. These findings highlight the challenges of studying drought impacts using passive manipulation, especially when precipitation patterns are highly variable. Overall, as droughts in the southwestern US are expected to become more frequent and intense due to climate change, our results suggest that desert ecosystems will face declines in perennial species and increased temporal variability, but unlike desert grasslands dominated by perennial grasses, systems dominated by annual species may be highly resilient following multi‐year severe droughts.

## AUTHOR CONTRIBUTIONS

TO and SLC conceived of the study and wrote the first draft of the manuscript. TO performed data analyses and data management. All authors contributed to implementation of the experiment, data collection, and editing of the manuscript.

## Supporting information


Data S1.
**Fig. S1.** Photos of drought treatment shelters at the experiment sites. (a) Granite Cove, (b) Molar Junction, (c) White Tank, (d) McDowell, (e) Sevilleta Mixed, and (f) Sevilleta Black.
**Table S1.** Mean annual precipitation and annual precipitation for the four treatment years for each of the six study sites. Annual precipitation defined as the precipitation from the 365 day prior to data collection.
**Table S2.** Results of RII for total vegetative cover, grass cover, forb cover, annual species cover, perennial species cover, species richness, species evenness, species gains, species losses, and rank change. Results are considered significant when the upper and lower boundary of the 95% confidence interval does not overlap 0. Significant results are bolded. NA denotes where not enough data were available to calculate RII. These results are visualized in Figs. 2 and 3.
**Table S3.** Model summaries of regressions of treatment over years of experiment with community metrics as the response variable (Metric column) for each site (Site column). Term column denotes model terms including the intercept, treatment, number of years of the experiment, and the interaction between treatment and experiment year. Estimate, standard error, degrees of freedom, and *P*‐values are reported respectively. Marginal *R* ^2^ (*R* ^2^m) and combined *R* ^2^ (*R* ^2^c) for each model are reported as well. Significant terms (*P* < 0.05) are bolded.
**Table S4.** Comparison of changes from pretreatment to year four between drought and control plots at each site. Positive estimate values mean that richness and evenness changed more positively in drought treatment plots and that rank abundance changed more in drought treatment plots than in control plots. Therefore, estimates for richness and evenness change could be positive even if those values decreased from pretreatment to year four as long as they decreased less in drought treatment plots than control plots. Bolded values indicate that change for drought and treatment plots was significantly different (*P* < 0.05) for the given site.
**Table S5.** Results of regression models testing the relationship between seasonal precipitation and community metrics. Metric column denotes which of three metrics was used as the response variable: total vegetative cover, species richness (SR), and species evenness. *R* ^2^m denotes the marginal *R* ^2^ of the mixed effects model and *R* ^2^c denotes the combined *R* ^2^.
