# Supplementary material for: Herbaceous plant communities respond more to seasonal precipitation than cumulative drought in the hot deserts of the United States
Source: Plant Biol (Stuttg). 2025 Aug 12;28(3):860–71. doi: 10.1111/plb.70083 (PMC13089593; doi:10.1111/plb.70083)
Supplement: Supplementary file 1 — Data S1. Fig. S1. Photos of drought treatment shelters at the experiment sites. (a) Granite Cove, (b) Molar Junction, (c) White Tank, (d) McDowell, (e) Sevilleta Mixed, and (f) Sevilleta Black. Table S1. Mean annual precipitation and annual precipitation for the four treatment years for each of the six study sites. Annual precipitation defined as the precipitation from the 365 day prior to data collection. Table S2. Results of RII for total vegetative cover, grass cover, forb cover, annual species cover, perennial species cover, species richness, species evenness, species gains, species losses, and rank change. Results are considered significant when the upper and lower boundary of the 95% confidence interval does not overlap 0. Significant results are bolded. NA denotes where not enough data were available to calculate RII. These results are visualized in Figs. 2 and 3. Table S3. Model summaries of regressions of treatment over years of experiment with community metrics as the response variable (Metric column) for each site (Site column). Term column denotes model terms including the intercept, treatment, number of years of the experiment, and the interaction between treatment and experiment year. Estimate, standard error, degrees of freedom, and P‐values are reported respectively. Marginal R 2 (R 2m) and combined R 2 (R 2c) for each model are reported as well. Significant terms (P < 0.05) are bolded. Table S4. Comparison of changes from pretreatment to year four between drought and control plots at each site. Positive estimate values mean that richness and evenness changed more positively in drought treatment plots and that rank abundance changed more in drought treatment plots than in control plots. Therefore, estimates for richness and evenness change could be positive even if those values decreased from pretreatment to year four as long as they decreased less in drought treatment plots than control plots. Bolded values indicate that change for drought and treat [file PLB-28-860-s001.docx]

SUPPLEMENTAL MATERIAL


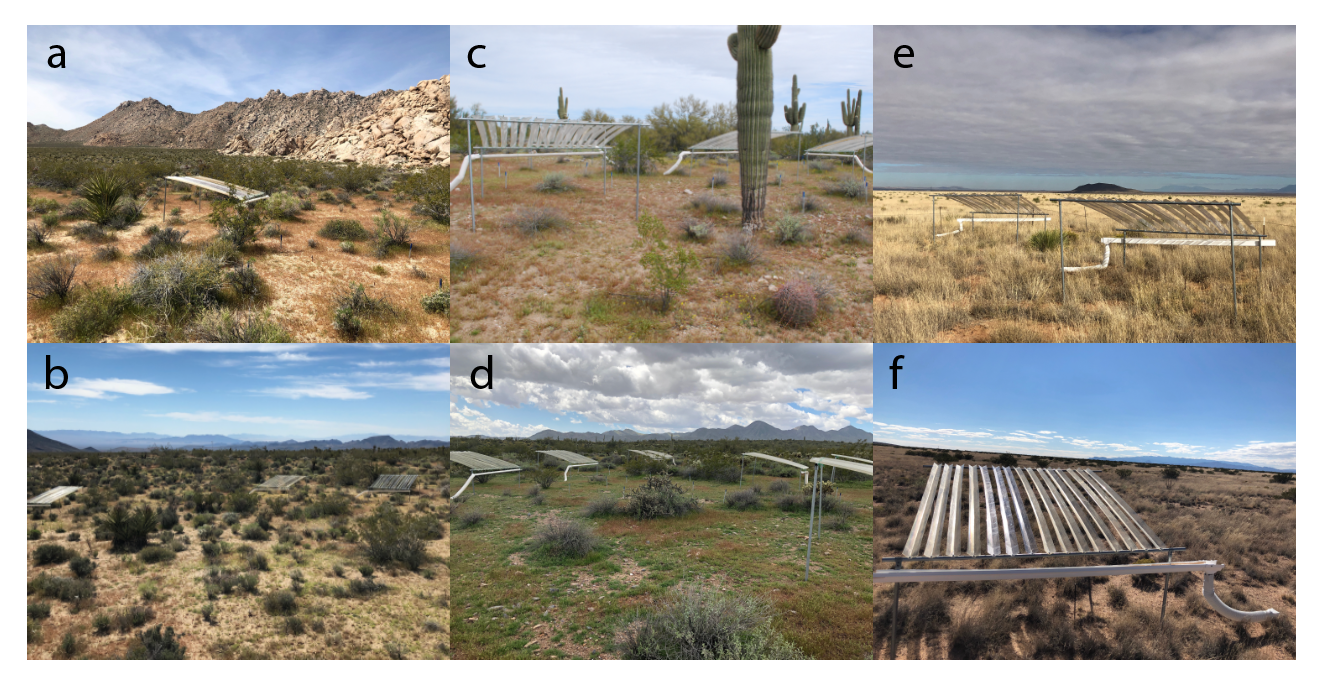


Figure S1. Photos of drought treatment shelters at the experiment sites. a) Granite Cove, b) Molar Junction, c) White Tank, d) McDowell, e) Sevilleta Mixed, and f) Sevilleta Black.

| Site | Desert | MAP (mm) | Annual precipitation – Year 1 | Annual precipitation – Year 2 | Annual precipitation – Year 3 | Annual precipitation – Year 4 |
| --- | --- | --- | --- | --- | --- | --- |
| Granite Cove | Mojave | 220 | 134 | 53 | 67 | 93 |
| Molar Junction | Mojave | 220 | 134 | 53 | 67 | 93 |
| White Tank | Sonoran | 212 | 342 | 203 | 201 | 324 |
| McDowell | Sonoran | 295 | 480 | 186 | 359 | 415 |
| Sevilleta Mixed | Chihuahuan | 249 | 248 | 269 | 254 | 201 |
| Sevilleta Black | Chihuahuan | 234 | 253 | 269 | 254 | 201 |

Table S1. Mean annual precipitation and annual precipitation for the four treatment years for each of the six study sites. Annual precipitation defined as the precipitation from the 365 day prior to data collection.

| Metric | Treatment year | Site | Mean RII | 95% lower | 95% upper |
| --- | --- | --- | --- | --- | --- |
| Cover | 1 | **Sevilleta Black** | **-0.47** | **-0.71** | **-0.23** |
|  |  | **Sevilleta Mixed** | **-0.52** | **-0.69** | **-0.34** |
|  |  | McDowell | 0.15 | -0.01 | 0.31 |
|  |  | White Tank | -0.35 | -0.74 | 0.04 |
|  |  | **Molar Junction** | **-0.23** | **-0.40** | **-0.06** |
|  |  | **Granite Cove** | **-0.31** | **-0.44** | **-0.18** |
|  | 2 | **Sevilleta Black** | **-0.52** | **-0.80** | **-0.24** |
|  |  | **Sevilleta Mixed** | **-0.23** | **-0.46** | **-0.001** |
|  |  | **McDowell** | **-0.94** | **-0.98** | **-0.89** |
|  |  | **White Tank** | **-0.87** | **-0.96** | **-0.78** |
|  |  | **Molar Junction** | **-0.96** | **-0.98** | **-0.94** |
|  |  | **Granite Cove** | **-0.98** | **-1.01** | **-0.96** |
|  | 3 | Sevilleta Black | -0.05 | -0.23 | 0.12 |
|  |  | Sevilleta Mixed | -0.17 | -0.35 | 0.02 |
|  |  | McDowell | -0.23 | -0.46 | 0.01 |
|  |  | **White Tank** | **-0.69** | **-0.79** | **-0.58** |
|  |  | **Molar Junction** | **-0.98** | **-1.02** | **-0.93** |
|  |  | **Granite Cove** | **-0.99** | **-0.99** | **-0.99** |
|  | 4 | Sevilleta Black | -0.38 | -0.86 | 0.11 |
|  |  | **Sevilleta Mixed** | **-0.32** | **-0.49** | **-0.14** |
|  |  | McDowell | 0.04 | -0.08 | 0.16 |
|  |  | White Tank | -0.03 | -0.21 | 0.15 |
|  |  | **Molar Junction** | **0.23** | **0.11** | **0.35** |
|  |  | Granite Cove | 0.02 | -0.23 | 0.26 |
|  |  |  |  |  |  |
| Annuals cover | 1 | **Sevilleta Black** | **-0.94** | **-1.00** | **-0.88** |
|  |  | **Sevilleta Mixed** | **-1.00** | **-1.00** | **-1.00** |
|  |  | **McDowell** | **0.23** | **0.07** | **0.39** |
|  |  | White Tank | -0.35 | -0.74 | 0.04 |
|  |  | **Molar Junction** | **-0.23** | **-0.40** | **-0.06** |
|  |  | **Granite Cove** | **-0.31** | **-0.44** | **-0.18** |
|  | 2 | **Sevilleta Black** | **-0.62** | **-1.08** | **-0.15** |
|  |  | **Sevilleta Mixed** | **-0.61** | **-1.14** | **-0.07** |
|  |  | **McDowell** | **-0.92** | **-0.98** | **-0.87** |
|  |  | **White Tank** | **-0.88** | **-0.97** | **-0.79** |
|  |  | **Molar Junction** | **-0.96** | **-0.98** | **-0.95** |
|  |  | **Granite Cove** | **-0.98** | **-1.00** | **-0.97** |
|  | 3 | **Sevilleta Black** | **0.26** | **0.09** | **0.44** |
|  |  | **Sevilleta Mixed** | **0.44** | **0.36** | **0.53** |
|  |  | **McDowell** | **-0.60** | **-0.88** | **-0.33** |
|  |  | **White Tank** | **-0.69** | **-0.79** | **-0.58** |
|  |  | **Molar Junction** | **-0.98** | **-1.02** | **-0.93** |
|  |  | **Granite Cove** | **-0.99** | **-0.99** | **-0.99** |
|  | 4 | Sevilleta Black | -0.25 | -0.84 | 0.34 |
|  |  | **Sevilleta Mixed** | **0.28** | **0.09** | **0.47** |
|  |  | McDowell | -0.07 | -0.34 | 0.20 |
|  |  | White Tank | -0.03 | -0.21 | 0.15 |
|  |  | **Molar Junction** | **0.23** | **0.11** | **0.36** |
|  |  | Granite Cove | 0.02 | -0.23 | 0.26 |
|  |  |  |  |  |  |
| Perennial cover | 1 | Sevilleta Black | -0.22 | -0.51 | 0.07 |
|  |  | **Sevilleta Mixed** | **-0.42** | **-0.62** | **-0.23** |
|  | 2 | **Sevilleta Black** | **-0.53** | **-0.76** | **-0.30** |
|  |  | Sevilleta Mixed | -0.20 | -0.47 | 0.07 |
|  | 3 | **Sevilleta Black** | **-0.85** | **-0.96** | **-0.75** |
|  |  | **Sevilleta Mixed** | **-0.70** | **-1.01** | **-0.39** |
|  | 4 | **Sevilleta Black** | **-0.68** | **-0.99** | **-0.36** |
|  |  | **Sevilleta Mixed** | **-0.78** | **-1.05** | **-0.52** |
|  |  |  |  |  |  |
| Species richness | 1 | Sevilleta Black | -0.16 | -0.42 | 0.10 |
|  |  | **Sevilleta Mixed** | **-0.18** | **-0.29** | **-0.07** |
|  |  | **McDowell** | **-0.09** | **-0.18** | **-0.01** |
|  |  | White Tank | -0.17 | -0.35 | 0.01 |
|  |  | Molar Junction | 0.02 | -0.09 | 0.12 |
|  |  | Granite Cove | -0.11 | -0.30 | 0.08 |
|  | 2 | Sevilleta Black | -0.06 | -0.19 | 0.07 |
|  |  | Sevilleta Mixed | -0.11 | -0.27 | 0.05 |
|  |  | **McDowell** | **-0.39** | **-0.64** | **-0.13** |
|  |  | White Tank | -0.22 | -0.46 | 0.01 |
|  |  | **Molar Junction** | **-0.56** | **-0.69** | **-0.43** |
|  |  | Granite Cove | -0.53 | -1.10 | 0.04 |
|  | 3 | Sevilleta Black | -0.01 | -0.16 | 0.14 |
|  |  | **Sevilleta Mixed** | **-0.31** | **-0.55** | **-0.07** |
|  |  | McDowell | -0.15 | -0.31 | 0.01 |
|  |  | White Tank | -0.08 | -0.18 | 0.03 |
|  |  | **Molar Junction** | **-0.75** | **-1.03** | **-0.47** |
|  |  | **Granite Cove** | **-0.72** | **-0.72** | **-0.72** |
|  | 4 | Sevilleta Black | -0.33 | -0.48 | 0.07 |
|  |  | **Sevilleta Mixed** | **-0.33** | **-0.42** | **-0.24** |
|  |  | **McDowell** | **0.25** | **0.12** | **0.37** |
|  |  | White Tank | -0.09 | -0.24 | 0.05 |
|  |  | **Molar Junction** | **0.13** | **0.04** | **0.22** |
|  |  | Granite Cove | -0.02 | -0.11 | 0.08 |
|  |  |  |  |  |  |
| Species evenness | 1 | Sevilleta Black | 0.03 | -0.12 | 0.17 |
|  |  | Sevilleta Mixed | -0.01 | -0.34 | 0.33 |
|  |  | **McDowell** | **-0.14** | **-0.25** | **-0.04** |
|  |  | White Tank | -0.04 | -0.10 | 0.03 |
|  |  | **Molar Junction** | **-0.24** | **-0.33** | **-0.15** |
|  |  | **Granite Cove** | **-0.39** | **-0.44** | **-0.34** |
|  | 2 | Sevilleta Black | 0.05 | -0.03 | 0.13 |
|  |  | Sevilleta Mixed | 0.02 | -0.12 | 0.15 |
|  |  | **McDowell** | **0.29** | **0.15** | **0.44** |
|  |  | **White Tank** | **0.38** | **0.11** | **0.66** |
|  |  | **Molar Junction** | **0.53** | **0.42** | **0.66** |
|  |  | **Granite Cove** | **0.56** | **0.56** | **0.56** |
|  | 3 | Sevilleta Black | 0.10 | -0.18 | 0.39 |
|  |  | Sevilleta Mixed | -0.07 | -0.34 | 0.21 |
|  |  | **McDowell** | **-0.15** | **-0.23** | **-0.07** |
|  |  | **White Tank** | **0.16** | **0.07** | **0.24** |
|  |  | Molar Junction | 0.20 | NA | NA |
|  |  | Granite Cove | NA | NA | NA |
|  | 4 | Sevilleta Black | 0.13 | -0.05 | 0.32 |
|  |  | Sevilleta Mixed | -0.04 | -0.24 | 0.14 |
|  |  | McDowell | -0.03 | 0.37 | 0.01 |
|  |  | **White Tank** | **-0.10** | **-0.14** | **-0.07** |
|  |  | **Molar Junction** | **-0.35** | **-0.37** | **-0.33** |
|  |  | **Granite Cove** | **-0.42** | **-0.48** | **-0.35** |

Table S2. Results of RII for total vegetative cover, grass cover, forb cover, annual species cover, perennial species cover, species richness, species evenness, species gains, species losses, and rank change. Results are considered significant when the upper and lower boundary of the 95% confidence interval does not overlap 0. Significant results are bolded. NA denotes where not enough data were available to calculate RII. These results are visualized in figures 2 and 3.

| **Metric** | **Site** | **term** | **estimate** | **SE** | **DF** | **p-value** | **R^2^ m** | **R^2^ c** |
| --- | --- | --- | --- | --- | --- | --- | --- | --- |
| Cover | Granite Cove | intercept | -9.3 | 5.0 | 36 | 0.07 | 0.24 | 0.24 |
|  |  | treatment (drought) | 11.7 | 7.6 | 12 | 0.15 |  |  |
|  |  | **experiment year** | **8.1** | **1.9** | **36** | **0.0001** |  |  |
|  |  | treatment * experiment year | -4.7 | 2.7 | 36 | 0.09 |  |  |
|  | Molar Junction | intercept | -3.1 | 3.4 | 36 | 0.38 | 0.29 | 0.29 |
|  |  | treatment | -0.33 | 4.9 | 12 | 0.95 |  |  |
|  |  | **experiment year** | **3.93** | **s1.3** | **36** | **0.004** |  |  |
|  |  | treatment * experiment year | 1.2 | 1.8 | 36 | 0.52 |  |  |
|  | White Tank | **intercept** | **25.0** | **7.7** | **40** | **0.0025** | 0.09 | 0.09 |
|  |  | treatment | -21.7 | 11.0 | 12 | 0.07 |  |  |
|  |  | experiment year | -1.3 | 2.9 | 40 | 0.65 |  |  |
|  |  | treatment * experiment year | 4.7 | 4.1 | 40 | 0.26 |  |  |
|  | McDowell | **intercept** | **26.8** | **9.1** | **40** | **0.005** | 0.001 | 0.001 |
|  |  | treatment | 0.34 | 12.9 | 12 | 0.98 |  |  |
|  |  | experiment year | 0.08 | 3.4 | 40 | 0.98 |  |  |
|  |  | treatment * experiment year | 0.37 | 4.8 | 40 | 0.94 |  |  |
|  | Sevilleta Mixed | **intercept** | **16.5** | **3.6** | **40** | **0.0000** | 0.50 | 0.66 |
|  |  | treatment | -5.7 | 5.0 | 12 | 0.28 |  |  |
|  |  | **experiment year** | **4.9** | **1.1** | **40** | **0.0001** |  |  |
|  |  | treatment * experiment year | -2.9 | 1.6 | 40 | 0.07 |  |  |
|  | Sevilleta Black | **intercept** | **19.0** | **4.6** | **40** | **0.0002** | 0.33 | 0.49 |
|  |  | treatment | -13.0 | 6.5 | 12 | 0.07 |  |  |
|  |  | **experiment year** | **4.6** | **1.5** | **40** | **0.004** |  |  |
|  |  | treatment * experiment year | -0.03 | 2.1 | 40 | 0.99 |  |  |
| Species richness | Granite Cove | **intercept** | **6.4** | **1.0** | **36** | **0.0000** | 0.23 | 0.23 |
|  |  | treatment | -2.4 | 1.5 | 12 | 0.12 |  |  |
|  |  | experiment year | -0.04 | 0.4 | 36 | 0.91 |  |  |
|  |  | treatment * experiment year | -0.04 | 0.5 | 36 | 0.94 |  |  |
|  | Molar Junction | **intercept** | **4.7** | **1.6** | **36** | **0.006** | 0.10 | 0.10 |
|  |  | treatment | -0.2 | 2.3 | 12 | 0.92 |  |  |
|  |  | experiment year | 1.1 | 0.6 | 36 | 0.06 |  |  |
|  |  | treatment * experiment year | -0.2 | 0.9 | 36 | 0.85 |  |  |
|  | White Tank | **intercept** | **5.6** | **0.9** | **40** | **0.0000** | 0.24 | 0.52 |
|  |  | treatment | -0.06 | 1.3 | 12 | 0.96 |  |  |
|  |  | **experiment year** | **1.0** | **0.3** | **40** | **0.001** |  |  |
|  |  | treatment * experiment year | -0.6 | 0.4 | 40 | 0.15 |  |  |
|  | McDowell | intercept | 2.3 | 1.2 | 40 | 0.06 | 0.36 | 0.36 |
|  |  | treatment | -1.1 | 1.7 | 12 | 0.52 |  |  |
|  |  | **experiment year** | **1.8** | **0.4** | **40** | **0.0003** |  |  |
|  |  | treatment * experiment year | 0.2 | 0.6 | 40 | 0.79 |  |  |
|  | Sevilleta Mixed | **intercept** | **4.4** | **0.6** | **40** | **0.0000** | 0.35 | 0.48 |
|  |  | treatment | -0.6 | 0.8 | 12 | 0.43 |  |  |
|  |  | experiment year | 0.05 | 0.2 | 40 | 0.77 |  |  |
|  |  | treatment * experiment year | -0.4 | 0.3 | 40 | 0.14 |  |  |
|  | Sevilleta Black | **intercept** | **3.6** | **0.5** | **40** | **0.0000** | 0.04 | 0.30 |
|  |  | treatment | -0.2 | 0.7 | 12 | 0.84 |  |  |
|  |  | experiment year | 0.1 | 0.2 | 40 | 0.60 |  |  |
|  |  | treatment * experiment year | -0.1 | 0.2 | 40 | 0.61 |  |  |
| Evenness | Granite Cove | **intercept** | **0.6** | **0.1** | **28** | **0.0001** | 0.10 | 0.10 |
|  |  | treatment | -0.2 | 0.2 | 12 | 0.38 |  |  |
|  |  | experiment year | -0.1 | 0.05 | 28 | 0.08 |  |  |
|  |  | treatment * experiment year | 0.03 | 0.1 | 28 | 0.72 |  |  |
|  | Molar Junction | **intercept** | **0.4** | **0.1** | **34** | **0.001** | 0.14 | 0.14 |
|  |  | treatment | 0.3 | 0.1 | 12 | 0.04 |  |  |
|  |  | experiment year | -0.02 | 0.04 | 34 | 0.62 |  |  |
|  |  | treatment * experiment year | -0.1 | 0.06 | 34 | 0.08 |  |  |
|  | White Tank | intercept | 0.1 | 0.07 | 40 | 0.08 | 0.08 | 0.08 |
|  |  | treatment | 0.2 | 0.1 | 12 | 0.13 |  |  |
|  |  | experiment year | 0.001 | 0.03 | 40 | 0.97 |  |  |
|  |  | treatment * experiment year | -0.04 | 0.04 | 40 | 0.36 |  |  |
|  | McDowell | **intercept** | **0.2** | **0.03** | **38** | **0.0000** | 0.06 | 0.12 |
|  |  | treatment | -0.01 | 0.04 | 12 | 0.77 |  |  |
|  |  | experiment year | -0.02 | 0.01 | 38 | 0.07 |  |  |
|  |  | treatment * experiment year | 0.003 | 0.01 | 38 | 0.82 |  |  |
|  | Sevilleta Mixed | **intercept** | **0.1** | **0.1** | **39** | **0.04** | 0.04 | 0.04 |
|  |  | treatment | 0.1 | 0.1 | 12 | 0.22 |  |  |
|  |  | experiment year | 0.01 | 0.02 | 39 | 0.67 |  |  |
|  |  | treatment * experiment year | -0.04 | 0.03 | 39 | 0.25 |  |  |
|  | Sevilleta Black | **intercept** | **0.1** | **0.03** | **36** | **0.0002** | 0.05 | 0.50 |
|  |  | treatment | 0.01 | 0.05 | 12 | 0.81 |  |  |
|  |  | experiment year | -0.01 | 0.01 | 36 | 0.21 |  |  |
|  |  | treatment * experiment year | 0.01 | 0.01 | 36 | 0.11 |  |  |

Table S3. Model summaries of regressions of treatment over years of experiment with community metrics as the response variable (Metric column) for each site (Site column). Term column denotes model terms including the intercept, treatment, number of years of the experiment, and the interaction between treatment and experiment year. Estimate, standard error, degrees of freedom, and p-values are reported respectively. Marginal R^2^ (R^2^m) and combined R^2^ (R^2^c) for each model are reported as well. Significant terms (p < 0.05) are bolded.

| Metric | Site | Estimate | SE | df | p-value |
| --- | --- | --- | --- | --- | --- |
| Richness change | Granite Cove | 0.2 | 0.1 | 71 | 0.07 |
|  | Molar Junction | -0.02 | 0.1 | 71 | 0.85 |
|  | White Tank | 0.2 | 0.1 | 71 | 0.11 |
|  | McDowell | 0.04 | 0.1 | 71 | 0.69 |
|  | **Sevilleta Mixed** | **0.4** | **0.1** | **71** | **0.002** |
|  | Sevilleta Black | 0.1 | 0.1 | 35 | 0.22 |
| Evenness change | Granite Cove | -0.01 | 0.1 | 69 | 0.94 |
|  | Molar Junction | 0.08 | 0.1 | 69 | 0.46 |
|  | White Tank | 0.01 | 0.1 | 69 | 0.88 |
|  | McDowell | -0.01 | 0.1 | 69 | 0.96 |
|  | Sevilleta Mixed | 0.16 | 0.1 | 69 | 0.13 |
|  | Sevilleta Black | -0.08 | 0.1 | 69 | 0.46 |
| Rank change | Granite Cove | 0.01 | 0.03 | 71 | 0.60 |
|  | Molar Junction | 0.03 | 0.03 | 71 | 0.31 |
|  | White Tank | 0.01 | 0.03 | 71 | 0.65 |
|  | McDowell | -0.01 | 0.03 | 71 | 0.74 |
|  | **Sevilleta Mixed** | **-0.06** | **0.03** | **71** | **0.04** |
|  | Sevilleta Black | -0.04 | 0.03 | 71 | 0.16 |

Table S4. Comparison of changes from pretreatment to year four between drought and control plots at each site. Positive estimate values mean that richness and evenness changed more positively in drought treatment plots and that rank abundance changed more in drought treatment plots than in control plots. Therefore, estimates for richness and evenness change could be positive even if those values decreased from pretreatment to year four as long as they decreased less in drought treatment plots than control plots. Bolded values indicate that change for drought and treatment plots was significantly different (p < 0.05) for the given site.

| **Metric** | **Site** | **slope** | **p-value** | | **R^2^m** | **R^2^c** |
| --- | --- | --- | --- | --- | --- | --- |
| **Cover** | **Granite Cove** | **0.43** | **<0.001** | **0.5** | | **0.5** |
| **Cover** | **Molar Junction** | **0.24** | **<0.001** | **0.36** | | **0.36** |
| **Cover** | **White Tank** | **0.14** | **<0.001** | **0.17** | | **0.17** |
| **Cover** | **McDowell** | **0.14** | **<0.001** | **0.24** | | **0.24** |
| **Cover** | **Sev Mixed** | **0.11** | **<0.001** | **0.37** | | **0.37** |
| **Cover** | **Sev Black** | **0.09** | **<0.001** | **0.16** | | **0.31** |
|  |  |  |  |  | |  |
| **SR** | **Granite Cove** | **0.01** | **0.01** | **0.07** | | **0.23** |
| **SR** | **Molar Junction** | **0.06** | **<0.001** | **0.34** | | **0.34** |
| SR | White Tank | -0.005 | 0.16 | 0.02 | | 0.3 |
| SR | McDowell | -0.01 | 0.11 | 0.04 | | 0.04 |
| SR | Sev Mixed | 0.004 | 0.12 | 0.03 | | 0.42 |
| **SR** | **Sev Black** | **0.01** | **0.02** | **0.07** | | **0.29** |
|  |  |  |  |  | |  |
| **Evenness** | **Granite Cove** | **-0.002** | **0.04** | **0.07** | | **0.07** |
| **Evenness** | **Molar Junction** | **-0.002** | **0.002** | **0.14** | | **0.14** |
| **Evenness** | **White Tank** | **-0.001** | **0.04** | **0.06** | | **0.06** |
| Evenness | McDowell | -0.0001 | 0.07 | 0.04 | | 0.04 |
| Evenness | Sev Mixed | -0.0002 | 0.43 | 0.01 | | 0.01 |
| Evenness | Sev Black | -0.0002 | 0.08 | 0.02 | | 0.48 |

Table S5. Results of regression models testing the relationship between seasonal precipitation and community metrics. Metric column denotes which of three metrics was used as the response variable: total vegetative cover, species richness (SR), and species evenness. R^2^m denotes the marginal R^2^ of the mixed effects model and R^2^c denotes the combined R^2^.
